# The whole truth and nothing but the truth: the need for full reporting of randomised trials

**DOI:** 10.1186/s13741-015-0017-9

**Published:** 2015-07-22

**Authors:** Rupert M. Pearse

**Affiliations:** Barts & The London School of Medicine and Dentistry, Queen Mary University of London, London, UK; Adult Critical Care Unit, Royal London Hospital, London, E1 1BB UK

**Keywords:** Randomised trials, Fluid therapy, Methods, Monitoring, Physiologic, Surgery

## Abstract

The use of cardiac output monitoring to guide fluid and inotropic therapy in surgical patients has remained a controversial topic for more than 40 years. The reasons for this are numerous and complex, but key amongst them is the interplay between poor research methodology and the likely selective reporting of randomised trials. In this issue of Perioperative Medicine, we find a very unusual report, one which describes a randomised trial stopped for futility after the recruitment of only a small proportion of the target patient sample (Jammer et al. Periop Med). The authors offer no statistical analysis of their findings but simply an explanation of what went wrong. On the face of it, this exercise would seem to offer little of value to the general reader. How can publication of the findings of an unsuccessful trial contribute to the evidence base on this topic? To understand this, we must delve a little deeper into the evidence and see how these trials were designed.

## Background

Cardiac output-guided haemodynamic therapy is a complex intervention consisting of one or more therapeutic agents (fluid and/or inotropes), a monitoring device, and a human who interprets the data provided by this device using an algorithm, adjusting therapy doses accordingly. Trials of complex interventions have a particular set of limitations, so much so that in the UK, the Medical Research Council has developed methodological guidance on the design of such trials [1]. However, the sad truth is that many previous randomised trials of cardiac output-guided haemodynamic therapy have been of very poor methodological design. The key weaknesses are small patient samples recruited in single centres, use of subjective clinical outcome measures with no observer blinding to study group allocation, and the provision of control group care according to alternative algorithms which may be better or worse than usual clinical practice. These limitations are easily found, even amongst the most recent randomised trials, but if we search reports from more than 15 years ago, we can find far more serious problems including a lack of clarity around which patients were randomised to which treatment, failure to include the outcomes of all trial participants in an ‘intention to treat’ analysis, and undeclared links to commercial partners. These problems do not affect every published trial but are frequent enough to fuel long running arguments between experts in the field and to undermine wider confidence in the evidence base. Doctors are left uncertain as to the clinical benefits and cost effectiveness of the treatment and may be disproportionately influenced by one or two trial reports, often because these reflect their own prejudicial views. It is important that we recognise that this evidence base is flawed and cannot justify any lasting change in practice towards the use of cardiac output monitoring. There are two key reasons why the publication of the findings of unfinished trials will help to resolve this problem.

## Discussion

For this particular intervention, futility of the trial is likely to be associated with futility of the treatment. The investigators struggled to identify enough eligible patients for their trial and struggled to ensure they received the trial intervention. If we do not publish the results of unfinished trials, we will bias reporting towards ‘positive’ trials and create the false impression of an overall clinical benefit. Alternatively, if the outcome data from such trials are publically available, they may still be included in systematic reviews even if not subjected to statistical testing in the primary report. This counters the effect of publication bias. The second reason is less scientific but probably more important. Confidence in the evidence base has been systematically undermined by long running arguments between academics, many of whom have one vested interest or another in cardiac output guided therapy being shown to work. Counterintuitively, the publication of a report which does nothing to flatter those of us who work in this field does not undermine wider confidence in the evidence base but proves to the reader that a full and frank disclosure is taking place. All is in the public domain, nothing is hidden from sight, and all the relevant information can be taken into account as we formulate a view of the evidence. Jammer and colleagues must be congratulated for placing their work in the public domain, and the journal editors should be applauded for publishing it.

## Conclusion

After 40 years and more than forty randomised trials, we remain uncertain about the perceived benefits of cardiac output-guided haemodynamic therapy. The findings of our own large multi-centre trial simply reinforce this uncertainty [2]. Only a very large multi-centre trial can provide the definitive answers we seek. Such a trial seems unlikely to happen, unless supported by both industry and major public funder(s). If this robust evidence cannot be provided, we should consider whether cardiac output monitoring adds value for our patients or is simply a distraction to the provision of high quality perioperative care.
